# The association of Alzheimer's disease and related dementias blood‐based biomarkers with depressive symptoms

**DOI:** 10.1002/alz.71007

**Published:** 2025-12-31

**Authors:** Julia R. Bacci, Joanne Ryan, Anne M. Murray, Zimu Wu, Robyn L. Woods, Michael Berk, Michelle M. Mielke

**Affiliations:** ^1^ Department of Epidemiology and Prevention Wake Forest University School of Medicine Winston‐Salem North Carolina USA; ^2^ School of Public Health and Preventive Medicine Monash University Melbourne Victoria Australia; ^3^ Division of Geriatric and Palliative Medicine, Department of Medicine Hennepin Healthcare Minneapolis Minnesota USA; ^4^ Berman Center for Outcomes and Clinical Research Hennepin Healthcare Research Institute Minneapolis Minnesota USA; ^5^ The Institute for Mental and Physical Health and Clinical Translation (IMPACT), School of Medicine Deakin University Geelong Victoria Australia

**Keywords:** Alzheimer's disease, Alzheimer's disease and related dementias, blood‐based biomarkers, depression, depressive symptoms, epidemiology, late life

## Abstract

**INTRODUCTION:**

Depressive symptoms are common in older adults and have been associated with the risk of Alzheimer's disease/Alzheimer's disease and related dementias (AD/ADRD), but the mechanisms and biomarkers underlying this association remain unclear.

**METHODS:**

We included baseline data from 11,947 non‐demented adults aged ≥ 70 years at enrollment in the Aspirin in Reducing Events in the Elderly (ASPREE) clinical trial. Linear regressions were used to examine cross‐sectional associations between AD/ADRD blood‐based biomarkers (BBMs) and baseline depressive symptoms. Interactions between sex or apolipoprotein E (*APOE*) ε4 carrier status and BBMs were examined.

**RESULTS:**

Higher glial fibrillary acidic protein (GFAP) was associated with higher depressive symptoms. We did not observe an association between amyloid beta 42/40 ratio, phosphorylated tau181, or neurofilament light chain with depressive symptoms; interactions between sex or *APOE* ε4 with depressive symptoms were not significant.

**DISCUSSION:**

In this large, community‐based cohort of older adults, plasma GFAP was associated with greater depressive symptoms.

**Highlights:**

- Plasma glial fibrillary acidic protein was associated with depressive symptoms.
- Neuroinflammation may underlie depressive symptoms in this group.
- Future research is needed to examine sex differences in this association.

## 1 BACKGROUND

Between 2025 and 2050, the number of individuals living with Alzheimer's disease and Alzheimer's disease and related dementias (AD/ADRD) in the United States is predicted to nearly double, increasing from 7.2 million to 12.7 million.^1^ Depressive symptoms are common in AD/ADRD, manifesting early in the disease and becoming more severe with disease progression.^2^ Several studies have reported associations between AD/ADRD pathologies and depressive symptoms, often prior to the emergence of cognitive symptoms.^3^, ^4^, ^5^, ^6^, ^7^ These findings suggest that early changes in AD/ADRD pathologies, specifically amyloid and tau, may be mechanisms underlying the association between depressive symptoms in late life and AD/ADRD, and that the emergence of depressive symptoms among older adults may be an early indication of underlying AD/ADRD pathology. Blood‐based biomarkers (BBMs) have emerged as more accessible in vivo markers of AD/ADRD pathologies, including amyloid beta (Aβ)42/40 ratio,^8^, ^9^, ^10^ phosphorylated tau (p‐tau)181,^11^, ^12^, ^13^, ^14^ neurofilament light chain (NfL),^15^, ^16^, ^17^ and glial fibrillary acidic protein (GFAP).^18^, ^19^

Studies of the association between BBMs or imaging biomarkers of AD/ADRD pathologies and late‐life depressive symptoms have yielded mixed results. Several studies have shown that depressive symptoms are associated with brain amyloid measured on positron emission tomography (PET) imaging;^3^, ^5^, ^6^ however, a recent meta‐analysis reported that this association was not replicated when using BBMs of amyloid pathology, including plasma Aβ42/40 ratio or p‐tau181.^20^ Further investigations of the association between AD/ADRD pathologies and depressive symptoms are necessary in larger, community‐based studies.

Neurodegeneration and neuroinflammation are non‐specific processes present in both depression and AD/ADRD, suggesting that depression and AD/ADRD may share common biological pathways and environmental risk factors.^2^, ^21^, ^22^, ^23^ Depressive symptoms have been consistently associated with neurodegeneration/neuroprogression, measured on volumetric magnetic resonance imaging (MRI), cognitive changes, or plasma NfL.^24^, ^25^, ^26^, ^27^, ^28^ In addition, depressive symptoms are associated with low‐grade systemic inflammation and increased levels of proinflammatory cytokines, which can activate astrocytes and contribute to neurodegeneration or neuroinflammation.^29^ Astrocytes perform multiple critical functions in the central nervous system, including regulation of the blood–brain barrier (BBB), modulation of glutamatergic neurotransmission, and coordination of neuroimmune responses.^30^ Astrocytic dysfunction can result in increased BBB permeability, glutamatergic toxicity, and synthesis of proinflammatory cytokines.^30^ Astrocytes constitutively express an intermediate filament, referred to as GFAP.^31^ Elevated plasma GFAP may indicate astrocytic damage or death resulting from astrogliosis or neuroinflammation.^31^ Although plasma GFAP has been associated with clinical depression,^32^ the relationship between GFAP and depressive symptoms is unclear.

**RESEARCH IN CONTEXT**

1. **Systematic review**: A comprehensive review of the literature was conducted using traditional databases, including PubMed and Google Scholar. Depressive symptoms are common in older adults and have been associated with both the risk for and symptoms of Alzheimer's disease/Alzheimer's disease and related dementias (AD/ADRD). However, the mechanisms and biomarkers underlying this association remain unclear.
2. **Interpretation**: Plasma glial fibrillary acidic protein (GFAP) was associated with depressive symptoms. These results suggest that neuroinflammation may be a mechanism underlying depressive symptoms.
3. **Future directions**: Future studies should examine this relationship longitudinally to determine the directionality of the association between plasma GFAP with depressive symptoms. It is also of interest to examine whether this association differs by sex.

Depressive symptoms have been shown to vary by sex and apolipoprotein E (*APOE*) ε4 carrier status. Women generally have a higher incidence of depression.^33^ One study reported greater longitudinal increases in depressive symptoms among *APOE* ε4 carriers compared to non‐carriers;^34^ however, another study reported no association between change in depressive symptoms and *APOE* ε4 carrier status.^35^ Given these conflicting results, it is of interest to examine whether sex and *APOE* ε4 carrier status impact the association between AD/ADRD BBMs and depressive symptoms. Indeed, a recent meta‐analysis reported that in subgroup analyses, plasma NfL was associated with depressive symptoms among women and *APOE* ε4 carriers, but not men or *APOE* ε4 non‐carriers.^20^ However, that study did not adjust for several key confounding factors, such as history of depression and antidepressant use. Because those with a later onset of depressive symptoms (i.e., those without a history of depression) may have different underlying mechanisms than those with a history of depression, it is important to consider prior history. Antidepressant use may also influence both depressive symptoms and AD/ADRD BBMs. Therefore, both factors should be included in models.

The current study examined the association of AD/ADRD BBMs and depressive symptoms at baseline in the Aspirin in Reducing Events in the Elderly (ASPREE) clinical trial, a large, community‐based study of older adults. We also examined whether the association between AD/ADRD BBMs and depressive symptoms differed by sex or *APOE* ε4 carrier status. We hypothesized that higher levels of AD/ADRD BBMs, specifically NfL and GFAP, would be associated with higher depressive symptoms, and that these associations would be stronger for women and *APOE* ε4 carriers compared to men and non‐carriers, respectively.

## 2 METHODS

### 2.1 Study population

Details on the ASPREE clinical trial and inclusionary/exclusionary criteria have been published previously.^36^ Briefly, ASPREE was a double‐blind, randomized, placebo‐controlled, primary prevention trial designed to assess the effect of daily low‐dose aspirin on several endpoints, including dementia, persistent physical disability, and death. Community‐dwelling older adults aged ≥ 65 years were recruited from multiple centers across Australia and the United States from 2010 to 2014. Participants completed annual study visits, which included the collection of demographic information, lifestyle factors, measures of cognitive function, depressive symptoms, physical function, medication use, and laboratory testing. Exclusionary criteria for enrollment in ASPREE included history of a cardiovascular disease event, diagnosed dementia or a score < 78 on the Modified Mini‐Mental State Examination, a disability that affected the ability to perform activities of daily living, or a condition likely to cause death within 5 years. Institutional review boards in the United States and Australia approved study protocols. All participants provided informed consent.

**FIGURE 1:**
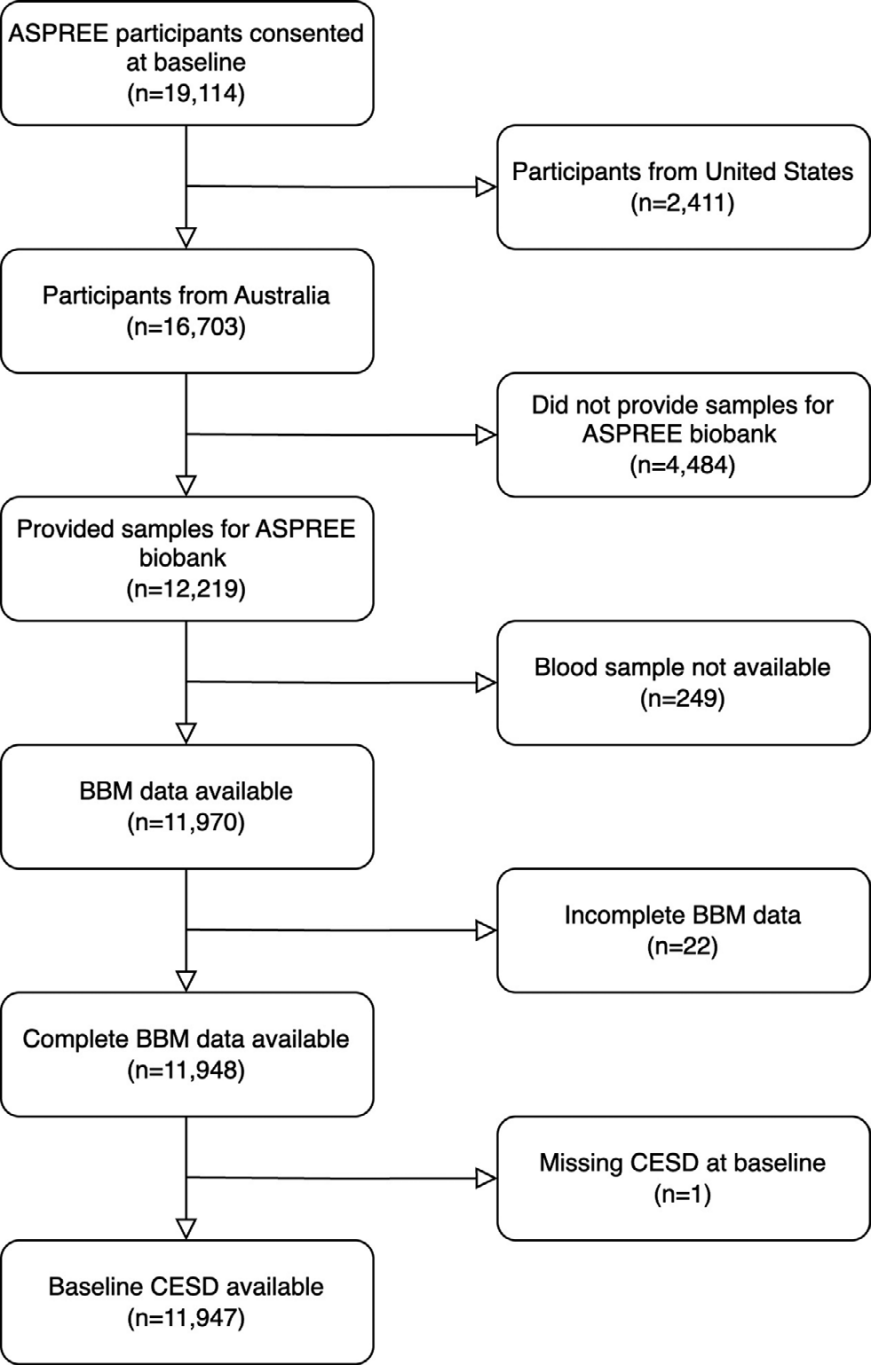
Flow chart for study participants. *Note*. Of the 19,114 participants consented at ASPREE baseline, 7167 participants were excluded due to missing or incomplete data, leaving 11,947 participants for the current study. ASPREE, Aspirin in Reducing Events in the Elderly; BBM, blood‐based biomarker; CESD, Center for Epidemiological Studies Depression.

In the current study, we used baseline visit data from Australian participants with AD/ADRD BBM and depressive symptom data. Of the 19,114 participants enrolled in the ASPREE clinical trial from 2010 to 2014, 7167 participants were excluded due to missing or incomplete data (Figure 1), leaving 11,947 participants for the current analyses. Compared to participants excluded from the current analyses, those included were less likely to be women (53.5% vs. 61.2%, *p *< 0.001), more likely to have < 12 years of education, had a higher proportion of White participants (98.7% vs. 84.8%, *p *< 0.001), and had lower levels of depressive symptoms (*p *= 0.001; data not shown). There were no significant differences in the age of those included and excluded (*p *= 0.054; data not shown).

### 2.2 AD/ADRD BBMs

As previously described, baseline non‐fasting blood samples were collected from Australian participants within the first 12 months of the trial.^37^ In 2023, aliquots were shipped from Australia to the Advanced Research and Diagnostic Laboratory (ARDL) at the University of Minnesota on dry ice to be assayed. Aβ42, Aβ40, NfL, and GFAP were quantified using the Simoa Human Neurology 4‐PlexE (N4PE) on the Quanterix HD‐X. The Simoa p‐Tau181 v2 assay was used to quantify p‐tau181 on the Quanterix HD‐X. Across 148 samples, the mean intra‐assay coefficients of variation (CVs) were 3.2% for Aβ40, 3.8% for Aβ42, 4.6% for GFAP, 4.6% for NfL, and 5% for p‐tau181. The mean inter‐assay CVs from 145 samples measured 4.8% for both Aβ40 and Aβ42, 6.1% for GFAP, 6.3% for NfL, and 6% for p‐tau181.

### 2.3 Depressive symptoms

Depressive symptoms were measured at baseline using the 10‐item version of the Center for Epidemiological Studies Depression Scale (CESD‐10).^38^ Participants responded to 10 items regarding how they felt or behaved over the past week on a four‐point scale that ranged from 0 (rarely or none of the time) to 3 (most of the time). The resulting scores range from 0 to 30, with higher scores indicating higher depressive symptoms.

### 2.4 Participant demographics and clinical characteristics

Participant demographics and lifestyle factors were self‐reported and included age, sex, race, education, living arrangements, smoking status, and alcohol consumption. Body weight, height, and waist circumference were measured by ASPREE study staff; weight and height were used to calculate body mass index (BMI). Antidepressant use was collected through medical record review and self‐report; participants were dichotomized as antidepressant users or antidepressant non‐users. Parental history of dementia was self‐reported; participants were dichotomized based on whether at least one parent had dementia. History of depression was self‐reported; participants were categorized as having a history of depression, no history of depression, or unsure of history of depression. Self‐report of a diabetes diagnosis, elevated glucose levels, or use of medications for diabetes were used to define the presence of diabetes. Prescriptions for medications for high blood pressure or measurement of high blood pressure (> 140/90 mmHg) at the baseline visit were used to define the presence of hypertension. Creatinine and hemoglobin values were derived from blood samples collected at baseline; age, sex, and creatinine were used to calculate estimated glomerular filtration rate (eGFR) using the Chronic Kidney Disease Epidemiology Collaboration (CKD‐EPI) equation.^39^ DNA extracted from baseline blood samples was genotyped to determine *APOE* ε4 carrier status; participants were dichotomized as *APOE* ε4 carriers based on whether at least one copy of the *APOE* ε4 allele was present. General global cognition was assessed using the Modified Mini‐Mental State Examination.^40^

### 2.5 Statistical analyses

Descriptive statistics for continuous variables are reported using median (range), and frequency (percentage) for categorical variables.

The association between AD/ADRD BBMs and depressive symptoms was examined using linear regression models. Log2 transformations were performed on BBMs prior to model entry due to non‐normal distributions. Using a model‐based approach, we conducted the analyses in three steps. We first examined the association between depressive symptoms and AD/ADRD BBMs in unadjusted models (Model 1). Model 2 adjusted for age, sex, race, education, parental history of dementia, *APOE* ε4 carrier status, living arrangements, smoking status, alcohol consumption, BMI, waist circumference, diabetes, hypertension, eGFR, hemoglobin, and general cognition (Model 2). Model 3 adjusted for all variables in Model 2, as well as antidepressant use and history of depression.

In additional analyses, we stratified Model 3 by sex and *APOE* ε4 carrier status to determine whether these factors modified the association between BBMs and depressive symptoms. These analyses were repeated, including an interaction term of BBMs and sex or *APOE* ε4 carrier status. For all statistical analyses, the threshold for significance was *p *< 0.05. The Benjamini–Hochberg procedure was used to adjust for multiple comparisons.^41^ Analyses were conducted in *R* 4.3.1.^42^

## 3 RESULTS

### 3.1 Sample characteristics

A total of 11,947 participants were included in this study. The median (range) age of participants was 73.9 (70.0–95.9) years; 53.5% of the participants were women (*n* = 6394); 98.6% were White. The median (range) CESD‐10 total score was 2.00 (0.0–30.0). Additional participant characteristics, lifestyle factors, chronic medical conditions, and medical histories are described in Table 1.

**TABLE 1:** Participant demographics.

Baseline characteristics	Total (*n* = 11,947)
Age (years)	73.87 (70.0–95.9)
Women (*n* [%])	6394 (53.5%)
Race (*n* [%])	
White	11782 (98.6%)
Non‐White	159 (1.3%)
Education (years)	12.00 (<9.0–21.0)
Parental history of dementia (*n* [%])	
Present	2653 (22.2%)
*APOE* ε4 carrier status (*n* [%])	
*APOE* ε4 carrier	2969 (24.9%)
Living arrangements (*n* [%])	
Alone	3727 (31.2%)
Smoking status (*n* [%])	
Current	379 (3.2%)
Former	4941 (41.4%)
Never	6627 (55.5%)
Alcohol consumption (*n* [%])	
Current	9523 (79.7%)
Former	559 (4.7%)
Never	1865 (15.6%)
BMI (*n* [%])	
14.0–20.9	402 (3.4%)
21.0–24.9	2656 (22.2%)
25.0–29.9	5446 (45.8%)
30.0–56.0	3395 (28.5%)
Waist circumference (*n* [%])	
Low	5475 (45.8%)
High	6381 (53.8%)
Diabetes (*n* [%])	
Present	1155 (9.7%)
Hypertension (*n* [%])	
Present	8898 (74.5%)
eGFR	74.02 (16.4‐111.1)
Hemoglobin	14.20 (11.0‐18.9)
General cognition	94.00 (78.0‐100.0)
Antidepressant use (*n* [%])	
Present	1341 (11.2%)
Absent	10606 (53.2%)
History of depression (*n* [%])	
Present	1098 (23.4%)
Absent	3457 (75.6%)
Unsure	45 (1.0%)
CESD total score	2.00 (0.0–30.0)
Aβ42/40 ratio	0.06 (0.01–0.36)
p‐tau181 (pg/mL)	31.46 (6.5–532.3)
NfL (pg/mL)	19.84 (2.7–187.3)
GFAP (pg/mL)	120.58 (19.0–1285.7)

*Note*: Continuous variables are presented as median (range). Categorical variables are presented as frequency (percentage).

Abbreviations: Aβ, amyloid beta; *APOE*, apolipoprotein E; BMI, body mass index; CESD, Center for Epidemiological Studies Depression; eGFR, estimated glomerular filtration rate; GFAP, glial fibrillary acidic protein; NfL, neurofilament light chain; pg/mL, picograms per milliliter; p‐tau, phosphorylated tau.

### 3.2 Association of BBMs with depressive symptoms

The associations of plasma Aβ42/40 ratio, p‐tau181, NfL, and GFAP with depressive symptoms are reported in Table 2. In unadjusted analyses (Model 1), lower p‐tau181, higher NfL, and higher GFAP levels were associated with higher depressive symptoms. After adjusting for variables in Model 2, the association between higher NfL or GFAP with higher depressive symptoms remained. In Model 3, after additionally adjusting for antidepressant use and history of depression, only higher GFAP remained associated with higher depressive symptoms, and the strength of the association more than doubled compared to Model 2. Across the three models, the results remained largely unchanged after correcting for multiple comparisons (Table 2).

**TABLE 2:** Relationship between AD/ADRD BBMs and depressive symptoms.

	Model 1 (*n* = 11,947)		Model 2 (*n *= 11,346)		Model 3 (*n* = 4428)	
	Beta (SE)	*p*	Beta (SE)	*p*	Beta (SE)	*p*
Aβ42/40 ratio	−0.034 (0.086)	0.690	−0.095 (0.089)	0.287	−0.182 (0.152)	0.231
p‐tau181	−0.096 (0.045)	0.033^*^, ^#^	−0.036 (0.047)	0.438	−0.032 (0.078)	0.682
NfL	0.183 (0.050)	<0.001^*^, ^#^	0.142 (0.060)	0.018^*^, ^#^	0.104 (0.103)	0.310
GFAP	0.221 (0.044)	<0.001^*^, ^#^	0.123 (0.051)	0.015^*^	0.270 (0.088)	0.002^*^, ^#^

*Note*: Model 1 linear regressions were unadjusted. Model 2 linear regressions were adjusted for age, sex, race, education, parental history of dementia, *APOE* ε4 carrier status, living arrangements, smoking status, alcohol consumption, BMI, waist circumference, diabetes, hypertension, eGFR, hemoglobin, and general cognition. Model 3 linear regressions additionally adjusted for antidepressant use and history of depression. In Model 1, lower p‐tau181, higher NfL, and higher GFAP were associated with higher depressive symptoms. In Model 2, the association between higher NfL or higher GFAP with higher depressive symptoms remained. In Model 3, only higher GFAP was associated with higher depressive symptoms.

Abbreviations: Aβ, amyloid beta; AD/ADRD, Alzheimer's disease/Alzheimer's disease and related dementias; *APOE*, apolipoprotein E; BBMs, blood‐based biomarkers; BMI, body mass index; eGFR, estimated glomerular filtration rate; GFAP, glial fibrillary acidic protein; NfL, neurofilament light chain; p‐tau, phosphorylated tau; SE, standard error.

* *p* < 0.05.

^#^ *p* < 0.05 after correction for multiple comparisons.

### 3.3 Effect of sex on the association of BBMs with depressive symptoms

In analyses stratified by sex, higher GFAP was only associated with higher depressive symptoms in the subsample of men (Table S1 in supporting information). In contrast, associations between Aβ42/40, p‐tau181, or NfL with depressive symptoms did not differ by sex. To formally test whether the association between BBMs and depressive symptoms differed by sex, we included BBM by sex interaction terms in Model 3. In these models, we observed a trending interaction of GFAP by sex (*p *= 0.075); however, none of the BBM by sex interaction terms were significant at the *p *< 0.05 level (Table 3; Table S1). The results remained unchanged after correcting for multiple comparisons.

**TABLE 3:** Interaction of sex and AD/ADRD BBMs on depressive symptoms.

	Beta (SE)	*p*
**Aβ42/40 ratio**		
Aβ42/40 ratio	−0.192 (0.234)	0.412
Sex	0.376 (1.230)	0.760
Aβ42/40 ratio x sex	0.016 (0.302)	0.958
**p‐tau181**		
p‐tau181	−0.041 (0.107)	0.704
Sex	0.209 (0.782)	0.789
p‐tau181 x sex	0.018 (0.153)	0.904
**NfL**		
NfL	0.181 (0.138)	0.189
Sex	0.938 (0.771)	0.224
NfL x sex	−0.148 (0.177)	0.402
**GFAP**		
GFAP	0.420 (0.122)	<0.001^*^, ^#^
Sex	2.178 (1.113)	0.050
GFAP x sex	−0.288 (0.162)	0.075

*Note*: Men were the reference group for sex. Linear regressions were adjusted for age, race, education, parental history of dementia, *APOE* ε4 carrier status, living arrangements, smoking status, alcohol consumption, BMI, waist circumference, diabetes, hypertension, eGFR, hemoglobin, general cognition, antidepressant use, and history of depression. The BBM by sex interaction terms were non‐significant.

Abbreviations: Aβ, amyloid beta; AD/ADRD, Alzheimer's disease/Alzheimer's disease and related dementias; *APOE*, apolipoprotein E; BBMs, blood‐based biomarkers; BMI, body mass index; eGFR, estimated glomerular filtration rate; GFAP, glial fibrillary acidic protein; NfL, neurofilament light chain; p‐tau, phosphorylated tau; SE, standard error.

* *p* < 0.05.

^#^ *p* < 0.05 after correction for multiple comparisons.

### 3.4 Effect of *APOE* ε4 on the association of BBMs with depressive symptoms

In analyses stratified by *APOE* ε4 carrier status, higher GFAP was associated with higher depressive symptoms only in the subsample of *APOE* ε4 non‐carriers (Table S2 in supporting information). This association was no longer significant after correction for multiple comparisons. In contrast, no significant associations were observed between Aβ42/40, p‐tau181, or NfL and depressive symptoms in either *APOE* ε4 subgroup. To formally test whether the association between BBMs and depressive symptoms differed by *APOE* ε4 carrier status, we included BBM by *APOE* ε4 carrier status interaction terms in Model 3. In these models, none of the BBM by *APOE* ε4 carrier status interaction terms were significant (Table 4; Table S2).

**TABLE 4:** Interaction of *APOE* ε4 carrier status and AD/ADRD BBMs on depressive symptoms.

	Beta (SE)	*p*
**Aβ42/40 ratio**		
Aβ42/40 ratio	−0.172 (0.177)	0.332
*APOE* ε4	−0.282 (1.418)	0.843
Aβ42/40 ratio x *APOE* ε4	−0.041 (0.345)	0.905
**p‐tau181**		
p‐tau181	−0.096 (0.089)	0.279
*APOE* ε4	−1.423 (0.909)	0.118
p‐tau181 x *APOE* ε4	0.263 (0.177)	0.138
**NfL**		
NfL	0.074 (0.113)	0.516
*APOE* ε4	−0.664 (0.913)	0.467
NfL x *APOE* ε4	0.133 (0.210)	0.527
**GFAP**		
GFAP	0.284 (0.101)	0.005^*^
*APOE* ε4	0.221 (1.246)	0.859
GFAP x *APOE* ε4	−0.052 (0.179)	0.772

*Note*: *APOE*: ε4 non‐carriers were the reference group. Linear regressions were adjusted for age, sex, race, education, parental history of dementia, living arrangements, smoking status, alcohol consumption, BMI, waist circumference, diabetes, hypertension, eGFR, hemoglobin, general cognition, antidepressant use, and history of depression. The BBM by *APOE* ε4 carrier status interaction terms were non‐significant.

Abbreviations: Aβ, amyloid beta; AD/ADRD, Alzheimer's disease/Alzheimer's disease and related dementias; *APOE*, apolipoprotein E; BBMs, blood‐based biomarkers; BMI, body mass index; eGFR, estimated glomerular filtration rate; GFAP, glial fibrillary acidic protein; NfL, neurofilament light chain; p‐tau, phosphorylated tau; SE, standard error.

* *p* < 0.05.

## 4 DISCUSSION

We examined the cross‐sectional associations of AD/ADRD BBMs (i.e., Aβ42/40 ratio, p‐tau181, NfL, GFAP) with depressive symptoms using a large community‐based sample of older adults. By using this well‐characterized cohort, we were able to adjust for known confounders, including medical comorbidities, cognitive function, antidepressant use, and history of depression. We also assessed whether sex and *APOE* ε4 carrier status modified these associations. In models adjusted for sociodemographic and lifestyle factors, chronic conditions, and general cognition, higher NfL or GFAP levels were associated with higher depressive symptoms. However, after additionally adjusting for antidepressant use and history of depression, only the association between plasma GFAP and depressive symptoms remained. Interactions between the BBMs and sex or *APOE* ε4 carrier status in relation to depressive symptoms were not significant.

### 4.1 Association of BBMs with depressive symptoms

#### 4.1.1 Aβ42/40 and p‐tau181

Studies examining the association between biomarkers of brain amyloid pathology and depressive symptoms have been inconsistent. Although some studies reported an association between brain amyloid measured via PET imaging and depressive symptoms among cognitively unimpaired older adults,^3^, ^5^, ^6^ other studies did not.^7^, ^43^ Notably, studies that reported significant associations used the PET radiotracer ^11^[C]‐Pittsburgh compound B (PiB) to image brain amyloid, while the other studies used ^18^[F] florbetapir. In the current study, we did not find associations between plasma Aβ42/40 ratio or p‐tau181 with depressive symptoms. These findings are consistent with a recent meta‐analysis that reported no association of the plasma Aβ42/40 ratio or p‐tau181 with depressive symptoms.^20^ Together, these results suggest that brain amyloid may not be the primary mechanism underlying depressive symptoms among older adults. However, as this was a cohort of cognitively unimpaired older adults, we cannot rule out that BBMs of brain amyloid may be associated with depressive symptoms at later stages of the disease. Indeed, a meta‐analysis reported an association between plasma amyloid and depressive symptoms in studies of participants with cognitive impairment, but not among cognitively unimpaired participants.^44^

#### 4.1.2 NfL

NfL is an established biomarker of large‐caliber axonal injury and neurodegeneration.^45^ A meta‐analysis found no association between NfL and depressive symptoms, but only adjusted for sociodemographic factors.^20^ In the present study, higher plasma NfL was associated with higher depressive symptoms in analyses adjusted for sociodemographic and lifestyle factors, chronic conditions, and general cognition. Notably, after additionally adjusting for a history of depression and antidepressant use, the association between NfL and depressive symptoms was attenuated. Thus, our findings indicate that levels of plasma NfL are not associated with current depressive symptoms, independent of disease history and treatment. However, because only a subset of participants had data on a history of depression and antidepressant use, we cannot rule out that this attenuation reflects the reduction in the sample size. Further research is necessary to understand differences in the mechanisms underlying depressive symptoms that emerge late in life compared to those that emerge earlier, and the effect of antidepressants, and type, on AD/ADRD BBMs and their association with depressive symptoms.

#### 4.1.3 GFAP

GFAP is an intermediate filament that is overexpressed by reactive astrocytes during brain inflammatory events. Elevated GFAP has been associated with brain amyloid deposition,^46^ and with depression.^32^ Notably, prior work reported that levels of GFAP are positively associated with age at the onset of depression among individuals aged 34 to 86 years.^47^ In the current study, we found an association between GFAP and depressive symptoms among older adults that remained after adjusting for sociodemographic and lifestyle factors, chronic conditions, general cognition, history of depression, and antidepressant use. These findings suggest that elevated GFAP may contribute to depressive symptoms among older adults and could be indicative of a shared mechanism underlying both depressive symptoms and AD/ADRD. However, because this study was cross‐sectional, we cannot rule out reverse causation, by which depressive symptoms, which are associated with systemic inflammation, may lead to elevations in GFAP.^29^, ^48^

### 4.2 Effect of sex or *APOE* ε4 on the association of BBMs with depressive symptoms

#### 4.2.1 Sex

Studies have shown that levels of plasma GFAP are higher among women compared to men.^49^, ^50^, ^51^ Although sex‐stratified analyses have found that GFAP is associated with AD/ADRD biomarkers and cognition among men, few studies have examined whether the association between plasma GFAP and depressive symptoms differs by sex.^52^ In our analyses stratified by sex, contrary to our hypothesis, we found that the association between GFAP and depressive symptoms was significant among men, not women, but the interaction term of GFAP by sex in relation to depressive symptoms was only trending. This finding is inconsistent with a recent meta‐analysis that reported no associations between plasma GFAP and depressive symptoms, among men or women.^20^ This discrepancy may be due to differences in sample characteristics, sample size, or the questionnaires used to measure depressive symptoms. In sex‐stratified analyses, the current study included 4428 Australian participants from the ASPREE clinical trial, which used the CESD‐10 to measure depressive symptoms, whereas the meta‐analysis included seven cohort studies from the Netherlands that used four different measures of depressive symptoms (*n* = 2355).^20^

#### 4.2.2 *APOE* ε4

Higher levels of plasma GFAP have been demonstrated both among *APOE* ε4 carriers and in individuals with depression.^32^, ^53^ Few studies have examined the association between plasma GFAP with depressive symptoms, and whether this association differs by *APOE* ε4 carrier status. A meta‐analysis found that higher levels of GFAP were associated with depressive symptoms among *APOE* ε4 non‐carriers in two cohorts when analyzing them separately; however, these cohorts had small sample sizes, and this association was attenuated in the pooled analysis that included seven cohorts.^20^ In our analyses stratified by *APOE* ε4 carrier status, the association between GFAP and depressive symptoms was significant only among non‐carriers; however, this association was no longer significant after adjusting for multiple comparisons. These findings suggest that among *APOE* ε4 non‐carriers, neuroinflammation may contribute to the occurrence of depressive symptoms; however, as the GFAP by *APOE* ε4 carrier status interaction term was not significant, these findings should be interpreted with caution.

### 4.3 Strengths and limitations

Strengths of our study include a large sample of community‐dwelling older adults and robust characterization of chronic conditions, medical histories, and concomitant medications, which enabled us to adjust for known confounders that may impact the association of BBMs with depressive symptoms.

However, there are limitations that also warrant consideration. First, the temporality of the relationship between AD/ADRD BBMs and depressive symptoms cannot be examined due to the cross‐sectional design. Second, at the time of publication, plasma p‐tau217 was not available in the ASPREE dataset. Although increasing evidence supports p‐tau217 as a superior BBM of amyloid pathology compared to p‐tau181, the non‐significant associations of both Aβ42/40 and p‐tau181 with depressive symptoms, coupled with the strong correlation between p‐tau181 and p‐tau217, suggest that the inclusion of p‐tau217 would likely not change our results.^14^ Third, the history of depression and antidepressant use was available for only a subset of participants (*n* = 4428), substantially reducing the sample size in Model 3. However, even within the stratified analyses, the study remained sufficiently powered to detect small effects, with detectable effect sizes ranging from 0.009 among the largest subgroup (*APOE* ε4 non‐carriers) to 0.028 among the smallest subgroup (*APOE* ε4 carriers). Fourth, the population was largely White, limiting the generalizability of our findings to more diverse populations.

## 5 CONCLUSION

In summary, we observed a cross‐sectional association of plasma GFAP, a biomarker of neuroinflammation, with depressive symptoms at baseline in a large sample of well‐characterized community‐dwelling older adults. In sex‐stratified analyses, this association was only observed among men, suggesting that neuroinflammation may be a mechanism underlying the occurrence of depressive symptoms in this subgroup. However, as the interaction term was non‐significant, this result needs to be investigated in additional and more diverse cohorts.

## CONFLICT OF INTEREST STATEMENT

Dr. Mielke has served on scientific advisory boards and/or has consulted for Acadia, Althira, Biogen, Eisai, Lilly, Merck, Neurogen Biomarking, Novo Nordisk, and Roche; received speaking honorariums from Novo Nordisk, PeerView Institute, and Roche; and receives grant support from the National Institutes of Health, Department of Defense, Alzheimer's Association, and Davos Alzheimer's Collaborative. Dr. Anne M. Murray receives grant support from the National Institutes of Health. Dr. Joanne Ryan serves as a non‐paid co‐chair for Dementia Australia Research Foundation scientific panel; received honoraria from the Victorian Coronial Council–Department of Justice and Community Service; and receives grant support from the National Institutes of Health, the National Health and Medical Research Council, the Medical Research Future Fund and the Australian Research Council. M. Berk reports grant funding from the Wellcome Trust, Medical Research Future Fund, Victorian Government Department of Jobs, Precincts and Regions, Janssen Lundbeckfonden Copenhagen, St. Biopharma, Milken Baszucki Brain Research Fund, Stanley Medical Research Institute, Danmarks Frie Forskningsfond Psykiatrisk Center Kovenhavn, Patient‐Centered Outcomes Research Institute (PCORI), Australian Eating Disorders Research and Translation Centre AEDRTC, USA Department of Defense Office of the Congressionally Directed Medical Research Programs (CDMRP), Equity Trustees Limited and Advisory boards including Janssen, Otsuka, St Biopharma, Actinogen, and Servier, all unrelated to this work. All other authors declare no conflicts of interest. Author disclosures are available in the supporting information.None of the authors has any conflicts of interest. Author disclosures are available in the supporting information.

## CONSENT STATEMENT

Study protocols were approved by institutional review boards at each participating institution across Australia and the United States. All participants provided informed consent.

## Supporting information




**Table S1**. Relationship between AD/ADRD BBMs and Depressive Symptoms Stratified by sex.
**Table S2**. Relationship between AD/ADRD BBMs and Depressive Symptoms Stratified by *APOE*4 Carrier Status.

Supporting Information
