# Supplementary material for: The association of Alzheimer's disease and related dementias blood‐based biomarkers with depressive symptoms
Source: Alzheimers Dement. 2025 Dec 31;22(1):e71007. doi: 10.1002/alz.71007 (PMC12756043; doi:10.1002/alz.71007)
Supplement: Supplementary file 1 — Table S1. Relationship between AD/ADRD BBMs and Depressive Symptoms Stratified by sex. Table S2. Relationship between AD/ADRD BBMs and Depressive Symptoms Stratified by APOE4 Carrier Status. [file ALZ-22-e71007-s001.docx]

**Supplementary Materials**

**Table S1**. Relationship between AD/ADRD BBMs and Depressive Symptoms Stratified by Sex.

|  | **Subsample of Women**  (n=2,428) | | **Subsample of Men**  (n=2,000) | | **AD/ADRD BBM * Sex Interaction Term** | |
| --- | --- | --- | --- | --- | --- | --- |
|  | **Beta (SE)** | ***p*** | **Beta (SE)** | ***p*** | **Beta (SE)** | ***p*** |
| Aβ42/40 ratio | -0.196 (0.207) | 0.344 | -0.151 (0.224) | 0.501 | 0.016 (0.302) | 0.958 |
| p-tau181 | -0.012 (0.117) | 0.919 | -0.044 (0.102) | 0.665 | 0.018 (0.153) | 0.904 |
| NfL | 0.016 (0.149) | 0.917 | 0.208 (0.141) | 0.140 | -0.148 (0.177) | 0.402 |
| GFAP | 0.133 (0.127) | 0.296 | 0.410 (0.121) | <0.001*^#^ | -0.288 (0.162) | 0.075 |

*Note.* Linear regressions were used to examine the association of AD/ADRD BBMs with depressive symptoms in sex-stratified subsamples. These models adjusted for age, race, education, parental history of dementia, *APOE*4 carrier status, living arrangements, smoking status, alcohol consumption, BMI, waist circumference, diabetes, hypertension, eGFR, hemoglobin, general cognition, antidepressant use, and history of depression. Analyses were repeated including an interaction term of AD/ADRD BBMs by sex; the *p*-values for interaction terms are reported. In sex-stratified analyses, higher GFAP was associated with higher depressive symptoms among men, but not women. The BBM by sex interaction terms were nonsignificant. **p* < 0.05; ^#^*p* < 0.05 after correction for multiple comparisons. Abbreviations: AD/ADRD, Alzheimer’s disease/Alzheimer’s disease and related dementias; BBMs, blood-based biomarkers; SE, standard error; Aβ, amyloid-beta; p-tau, phosphorylated tau; NfL, neurofilament light; GFAP, glial fibrillary acidic protein; *APOE*4, apolipoprotein E 4.

**Table S2**. Relationship between AD/ADRD BBMs and Depressive Symptoms Stratified by *APOE*4 Carrier Status.

|  | **Subsample of APOE4 Carriers**  (n=1,096) | | **Subsample of APOE4 Non-carriers**  (n=3,332) | | | **AD/ADRD BBM * APOE4 Carrier Status Interaction Term** | |
| --- | --- | --- | --- | --- | --- | --- | --- |
|  | **Beta (SE)** | ***p*** | **Beta (SE)** | ***p*** | | **Beta (SE)** | ***p*** |
| Aβ42/40 ratio | -0.226 (0.300) | 0.452 | -0.168 (0.176) | | 0.342 | -0.041 (0.345) | 0.905 |
| p-tau181 | 0.171 (0.159) | 0.282 | -0.104 (0.089) | | 0.244 | 0.263 (0.177) | 0.138 |
| NfL | 0.288 (0.217) | 0.184 | 0.056 (0.117) | | 0.631 | 0.133 (0.210) | 0.527 |
| GFAP | 0.234 (0.172) | 0.174 | 0.280 (0.103) | | 0.007* | -0.052 (0.179) | 0.772 |

*Note.* Linear regressions were used to examine the association of AD/ADRD BBMs with depressive symptoms in subsamples stratified by *APOE*4 carrier status. These models adjusted for age, sex, race, education, parental history of dementia, living arrangements, smoking status, alcohol consumption, BMI, waist circumference, diabetes, hypertension, eGFR, hemoglobin, general cognition, antidepressant use, and history of depression. Analyses were repeated including an interaction term of AD/ADRD BBMs by *APOE*4 carrier status; the *p*-values for interaction terms are reported. In analyses stratified by *APOE*4 carrier status, higher GFAP was associated with higher depressive symptoms only among *APOE*4 non-carriers. The BBM by *APOE*4 carrier status interaction terms were nonsignificant. **p* < 0.05. Abbreviations: AD/ADRD, Alzheimer’s disease/Alzheimer’s disease and related dementias; BBMs, blood-based biomarkers; SE, standard error; Aβ, amyloid-beta; p-tau, phosphorylated tau; NfL, neurofilament light; GFAP, glial fibrillary acidic protein.
